# Conversion of the Sensor Kinase DcuS to the Fumarate Sensitive State by Interaction of the Bifunctional Transporter DctA at the TM2/PAS_C_-Linker Region

**DOI:** 10.3390/microorganisms9071397

**Published:** 2021-06-28

**Authors:** Marius Stopp, Christopher Schubert, Gottfried Unden

**Affiliations:** Institute for Molecular Physiology, Johannes Gutenberg-University, 55122 Mainz, Germany; stopp@uni-mainz.de (M.S.); schubert@uni-mainz.de (C.S.)

**Keywords:** sensor kinase DcuS, bifunctional transporter DctA, sensor complex, structural co-regulator

## Abstract

The membrane-bound C_4_-dicarboxylate (C4DC) sensor kinase DcuS of *Escherichia coli* typically forms a protein complex with the C4DC transporter DctA. The DctA × DcuS complex is able to respond to C4DCs, whereas DcuS without DctA is in the permanent ON state. In DctA, the C-terminal helix 8b (H8b) serves as the site for interaction with DcuS. Here the interaction site in DcuS and the related structural and functional adaptation in DcuS were determined. The Linker connecting transmembrane helix 2 (TM2) and the cytosolic PAS_C_ (Per-ARNT-SIM) domain of DcuS, was identified as the major site for interaction with DctA-H8b by in vivo interaction studies. The Linker is known to convert the piston-type transmembrane signaling of TM2 to a tilting motion which relies on a resolution of the Linker-Linker’ homodimer in the presence of C4DCs. Absence of DctA caused decreased cross-linking in the Linker, as identified by oxidative Cys-cross-linking. This response resembled structurally and functionally that of fumarate activation in the DctA × DcuS complex. Overall, formation of the DctA × DcuS complex is based on the interaction of the DcuS Linker with DctA H8b; the interaction is required to set DcuS in the C4DC-responsive state by stabilizing the linker-linker’ homodimer in DcuS. This work identifies DctA as a structural co-regulator of DcuS sensor kinase.

## 1. Introduction

Many membrane-anchored bacterial histidine kinases recognize ambient cues through extra-cytoplasmic sensor domains [1,2]. For the cellular response, the signal is transmitted across the membrane, resulting in phosphorylation of the cytosolic kinase domain and subsequently of the response regulator that controls typically transcriptional expression of target genes. The fumarate, or C_4_-dicarboxylate (C4DC), sensor kinase DcuS of the DcuS-DcuR two-component system of *Escherichia coli* consists of PAS_P_ (Per-ARNT-SIM), a transmembrane region composed of two antiparallel transmembrane helices TM1 and TM2, a cytoplasmic PAS_C_ and the kinase domains [3,4,5]. The sensory PAS_P_ domain is framed by TM1 and TM2 and is located in the periplasm. DcuS forms a homo-dimer in the bacterial membranes [6]. Transmembrane signaling is initiated by binding of a C4DC ligand to PAS_P_ [7,8,9] which results in compaction of the binding site and triggers an upward movement of the C-terminal α6-helix of PAS_P_. This displacement of PAS_P_ α6 has been shown for the orthologous CitA citrate sensor kinase [10,11]. The uplift pulls TM2 of DcuS in the direction of the periplasm in a piston-like movement [12,13]. The piston movement is affected by the TM2-TM2′ homodimer that ranges from the C-terminal α6-helix of PAS_p_ on the periplasmic side to the TM2/PAS_C_-Linker on the cytosolic side [13] which comprises residues C_199_ILVKVLKKILFG_211_. In the TM2/PAS_C_-Linker the stable homodimer is partially resolved or rearranged in the presence of fumarate to initiate signal conversion that controls kinase function [13]. Thus, the TM2/PAS_C_-Linker converts the piston movement of the TM2/TM2′ dimer and establishes a tilting motion for signal transmission to PAS_C_. In contrast to TM2, the position of TM1 shifted only slightly upon activation of DcuS [12].

DcuS therefore contains all domains and properties required for its function as a TM sensor kinase, including stimulus perception, TM signaling, signal conversion and transfer to the kinase domain. Expression of DcuS-DcuR regulated genes such as *dctA* and *dcuB* encoding the aerobic and the anaerobic C4DC transporters DctA and DcuB becomes constitutive, however, when either DctA or DcuB are deleted [14,15]. It was shown that DcuS is converted to the permanent ON state in the absence of DctA or DcuB, regardless of the availability of fumarate. On the other hand, the fumarate responsive state of DcuS is only formed when DcuS is present as a DctA × DcuS or DcuB × DcuS complex [15,16]. Thus, the basal form of DcuS is the active form of the protein which has to be converted by the transporters to the fumarate responsive form. DctA interacts by its short C-terminal amphipathic helix 8b (H8b) with DcuS, and deletion, truncation, or mutation of H8b renders DctA incompetent for interaction [16]. The co-regulation by DctA is independent of the transport activity of the transporter [17], and the C4DC binding site in DctA [17,18]. The data led to a model for a DctA × DcuS sensor complex in which DcuS serves as the sensor and DctA as a component for assembling an intact sensor [5,17]. Overall, DctA is a bifunctional protein that serves primarily as a transporter for C4DCs and secondarily as a co-regulator of the DcuS sensor. Under anaerobic conditions when DcuB takes over the role of C4DC transport from DctA [19,20], DcuB adopts the function as the co-regulator of DcuS [15,18].

The DctA site for interaction with DcuS has been identified as α-helix H8b. H8b represents a short amphipathic helix 2.1that follows transmembrane helix TM8 on the cytoplasmic side of the membrane. H8b represents the C-terminus of DctA and is not present in DctA orthologs [21]. In DcuS, the cytosolic portions of the protein play the major role in the interaction with DctA [21]. The present experiments aim to identify the interaction site of DcuS in more detail and to obtain clues on how the interaction with DctA converts DcuS to the C4DC responsive state. The studies narrowed down the interaction site to PAS_C_ and in particular to the TM2/PAS_C_-Linker. The role of the linker in the interaction and conversion of DcuS to the regulatory competent state was tested by analyzing the role of DctA in the structural arrangement of the DcuS linker. H8b and the TM2/PAS_C_-linker region was subject to structural modeling for more information and clues on the interaction of DcuS with DctA, which will pave the way for detailed interaction studies at this site and their functional consequences.

## 2. Materials and Methods

### 2.1. Bacterial Strains and Growth Conditions

The *E. coli* K12 strains and the plasmids are listed in Table S1. All molecular methods, cloning, phage P1 transduction, DNA isolation, and manipulations were performed according to standard procedures [15,22,23,24,25]. Phage P1 transduction to obtain IMW660 was performed as described previously [22,26]. The recipient strain was C43 and the donor strain of the inactivated *dctA* and *dcuS* gene via Kan^R^ resistance were the Keio collection strains JW3496 and JW4086. The Kan^R^ resistance cassette is flanked by an *flp* recombinase site so that Kan^R^ can be removed via the thermolabile pCP20 plasmid. The success of recombination was verified by sequencing the corresponding region. Bacteria were grown aerobically at 37 °C in lysogeny broth (LB) as indicated. All media were inoculated at 37 °C with 1–5% (*v*/*v*) of an overnight culture grown under the same conditions and in the same medium. When required, antibiotics were added as follows: 50 µg mL^−1^ kanamycin and 100 µg mL^−1^ ampicillin.

### 2.2. BACTH and β-galactosidaseAassay

The cloning of DcuS fragments to T18 and T25 for bacterial two-hybrid (BACTH) experiments was performed as described [12]. Bacteria (Table S1) for the BACTH measurements were grown aerobically in LB medium. Interactions in the BACTH system were measured in terms of the β-galactosidase activity [22]. BACTH experiments and measurement of β-galactosidase activity were conducted as described previously [24] with slight modifications [18]. A volume of 250 µL per well was used for photometric measurements. In addition, cells were permeabilized by adding 200 µL of culture to 800 µL β-galactosidase buffer (KPi buffer (100 mM potassium phosphate, pH 7.0), potassium chloride (10 mM), magnesium chloride (1 mM), cetyltrimethylammonium bromide (0.005% *w*/*v*), sodium deoxycholate (0.0025% *w*/*v*)) with dithiothreitol (DTT, 8 mM). For the β-galactosidase assay 150 mL of permeabilized cells were transferred to 96 well plates and incubated at 30 °C. To start the reaction 30 µL of *ortho*-nitrophenyl-β-D-galactoside (4 mg/mL) were added. The reaction was stopped after 20 min by addition of 70 µL Na_2_CO_3_ (1 M). Each strain was measured in 2 to four biological repeats and four independent samples each.

### 2.3. Time-Resolved Oxidative Cys-Cross-Linking

For the time-resolved Cys cross-linking (CL) *E. coli* C43(DE3) or IMW660 (Table S1) were transformed with plasmid pMW336 derivatives encoding DcuS single-Cys variants. After 2 h of growth DcuS expression was induced with 1 mM IPTG. and after reaching an OD of 2.5–3 100 µL of the cell suspension and the oxidant Cu (II)-1,10-phenanthrolie were mixed with 100 mL (for the reaction conditions see below). Incubation took place in 48-well plates with vigorous shaking (1050 rpM, 10 min) [12]. The reaction was stopped with 12.5 mM EDTA and 12.5 mM *N*-ethylmaleimide after 0.5, 3, 10, and 20 min and the samples were applied to non-reducing SDS-PAGE (gradient gels, 5–12%) and semi-dry Western blot. DcuS and its CL products were detected with anti-serum targeting the DcuS PAS_P_ domain and a peroxidase-coupled secondary antibody. Chemiluminescence detection was conducted with WesternBright™ ECL/peroxide solution (Advansta) and was visualized on X-ray films [13]. Image evaluation was performed with ImageJ software [13]. Reaction conditions with regards to oxidizing agent concentrations and temperatures for the respective DcuS single-Cys variants are the following: for DcuS variants G194C, L208C, L209C and R224C 0.5 mM CuSO_4_, 1.625 mM 1,10-phenanthroline, and 4 °C; for variant G190C 2 mM CuSO_4_, 7.5 mM 1,10-phenanthroline, and 4 °C compare [13]. 

### 2.4. In Silico Analysis

DcuS was modeled as a composition of the structures of PAS_P_, PAS_C_, and modelled TM2 and the linker from CL studies. UCSF Chimera ([25], version 1.13.1, build 41955) was used to fuse the structures obtained by homology modeling (PAS_P_, PAS_C_) and predicted structures (TM2, linker). The structure of the apo PAS_P_ monomer was obtained using the structure of DcuS homolog CitA as a template ([10], PDB ID: 2V9A). The PAS_C_ dimer structure was modeled using the structure of CitA as a template ([11], PDB ID: 5FQ1). The helix dimer from PAS_P_ to PAS_C_ was derived by structural analysis of the C-terminal region of PAS_P_ (α6) and the *N*-terminal region of PAS_C_ (α1) [9,27,28], and the CL data [12,13]. The cytoplasmic *N*-terminal coil, TM1, and the TM2-PAS_C_ linker were predicted as single subdomains by the server I-TASSER [29]. The kinase domain was modeled by I-TASSER, using the structurally similar kinase domain HK853 of *Thermotoga maritima* ([29,30], PDB ID: 4JAU). Multiple-sequence alignments (MSA) were performed with Unipro UGENE v34.0 ([31], 64-bit version, Mar 19 2020). For the MSA’s the default setting were used with the MUSCLE algorithm [32]. The primary sequences for DcuS and DctA were obtained through Uniprot [31]: *Escherichia coli*, DcuS (Uniprot ID: P0AEC8), CitA (P77510), and DctA (P0A830); *Klebsiella pneumoniae*, DcuS (A6T8R1), CitA (P52687), and DctA (A6TFD1); *Salmonella enterica*, DcuS (A0A3Z5XF95), CitA (A0A0D6H5A2), and DctA (A0A3V0F2H1); *Enterobacter cloacae*, DcuS (A0A156CKF7), CitA (A0A144N960), and DctA (A0A156JRS4); *Serratia marcescens*, DcuS (A0A221FN95), CitA (A0A3E2EAR4), and DctA (A0A0A5LIU9); *Dickeya dadantii*, DcuS (E0SEQ6), CitA (E0SAP8), and DctA (E0SES9).

## 3. Results

### 3.1. The TM2/PAS_C_-Linker of DcuS Plays an Important Role for the Interaction with H8b of DctA

A DcuS construct deficient for PAS_C_-Kin is known to lose most of the interaction with DctA and H8b whereas deletion of the kinase domain alone causes no significant decrease [16,21]. Therefore, PAS_C_ is important for interaction with DctA, in contrast to TM2 and PAS_P_. The earlier study did not address, however, the role of the TM2/PAS_C_-Linker (C_199_ILVKVLKKILFG_211_) since the constructs used were truncated within the Linker at position Lys206. 

**Figure 1:**
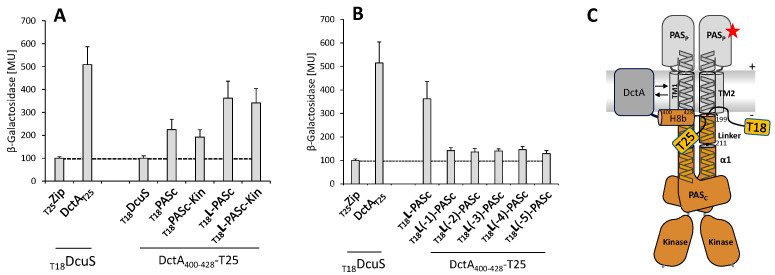
Interaction of PAS_C_ variants (with and without the TM2/PAS_C_-Linker) of DcuS with helix 8b of DctA tested in vivo with the bacterial two-hybrid system. (**A**) Test for the interaction of helix 8b (DctA_400-428_) with PAS_C_ or PAS_C_-Kin with and without Linker (L: I_200_LVKVLKKILFG_211_). (**B**) Test for Linker length in L-PAS_C_ interaction with helix 8b of DctA. L stands for full-length linker, L-1 (and so on) for a linker deleted for the first (and so on) *N*-terminal amino acid residue. *E. coli* BTH101(Δ*cyaA*) cultures were co-transformed with two plasmids that encoded proteins fused with the T25 and T18 domains of *Bordetella pertussis* adenylate cyclase. T18 was fused *N*-terminally to PAS_C_ variants, T25 C-terminally to DctA_400-428_ (**C**). β-Galactosidase activities are shown in Miller units (MU). The pair DcuS_T18_/_T25_Zip pair served as the negative control to indicate background β-galactosidase activity, and the pair DctA-T25 × T18-DcuS as a positive control. The BACTH assays in (**A**,**B**) were performed in three biological replicates with three repeats each. (**C**) shows a schematic presentation of DcuS, DctA in the membrane, and the respective domains to illustrate the sites of T18- and T25-fusions, respectively. The red star (fumarate) denotes binding of fumarate to one of the PAS_C_ domains, + and − represent the positive (periplasmic) and negative (cytoplasmic) sides of the membrane.

For domain interaction studies in vivo, a bacterial two-hybrid (BACTH) system was used that allows studies on individual domains, particularly the TM2/PAS_C_ Linker and the PAS_C_ domain. On the DctA side, helix H8b was fused to the T25 domain of the *Bordetella pertussis* adenylate cyclase, and for DcuS, the TM2/PAS_C_-Linker was combined with the T18 domain of the cyclase (Figure 1). The Linker was used in fusion with the cytosolic PAS_C_ domain to provide a suitable structural context for the short peptide (Figure 1A). The background or control for interaction was given by the activity of non-interacting proteins (DcuS × Zip) with approx. 100 MU, and full-length DcuS and DctA as a positive control with 515 MU. H8b alone was not able to interact with full-length DcuS when it is fused to T25 (Figure 1A) which can be due to structural constraints from T25. The H8b construct interacted, however, with PAS_C_ at significant levels (44.2% of the positive control), and the interaction was stimulated further (71.1% of the positive control) when the TM2/PAS_C_-Linker was present at the *N*-terminal end of PAS_C_. When PAS_C_ was replaced by a PAS_C_-Kin protein, interaction was similar to the PAS_C_ constructs supporting earlier data [16] that the kinase domain does not contribute to the interaction with DctA. Therefore, H8b of DctA interacts with PAS_C_, and the interaction is largely stimulated when the TM2/PAS_C_-Linker is present.

For more information on the TM2/PAS_C_-Linker in the interaction, the Linker was sequentially deleted by single amino acid residues starting from Ile200 at the *N*-terminal end (Figure 1B). After deletion of the first residue, the interaction dropped to a level similar to the Linker deficient state of PAS_C_. The interaction remained at the decreased level after deleting the following residues sequentially up to the fifth residue (Figure 1B) and further to the complete deletion of the Linker (not shown). The data indicate that either Leu200, or the length of the linker, is important for interaction. When Leu200 is replaced by Cys or Ala residues (Stopp et al., 2021), the fumarate response of DcuS in the expression of DcuS regulated genes is not affected. Even substitution of Leu200 by an Arg residue caused a loss of only 6% of wild-type activity of DcuS. Therefore, the Linker has the capacity to stimulate interaction with H8b when attached to PAS_C_, but the length of the Linker is critical. Deletion of residue Ile200 or of a full helical turn (4 residues) abolishes the interaction in the same way as full deletion of the Linker, whereas the type of the residue in position 200 is not critical.

When the linker region was studied by Ala replacement mutations, four of the mutations are conspicuous [13]. Replacement mutations K206A and V202A were ON or partial ON mutations, respectively, whereas L209A and F210A replacements resulted in full or partial loss of activity in the stimulation of DcuS dependent genes. Moreover, replacements of L201 (OFF), L202 (ON) and L205 (OFF) by Arg produce ON or OFF phenotypes depending on the position (not shown). Overall, at least six out of the 13 residues of the linker contribute to DcuS function in a preliminary assay, stressing the functional significance of the linker.

### 3.2. Absence of DctA Affects Cys-Cross-Linking Efficiency in the TM2/PAS_C_-Linker of DcuS

**Figure 2:**
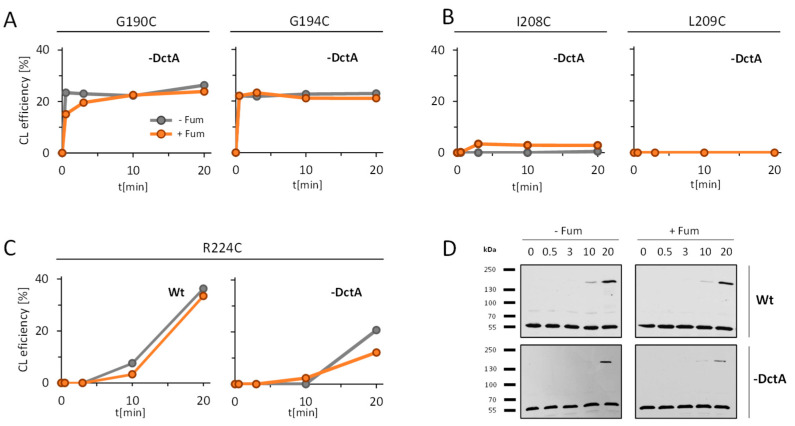
Time-resolved oxidative Cys CL for Cys variants located in (**A**) TM2 (G190C, G194C), (**B**) the TM2/PAS_C_-Linker (I208C, L209C), and (**C**) α1-PAS_C_ (R224C) in *E. coli* strain IMW660 deficient of *dctA* and *E. coli* C43(DE3) (wildtype for DctA, Wt). (**D**) Exemplary Western blots for CL of DcuS(R224C) in *E. coli* C43((DE3) (Wt, top) or IMW660 (-DctA, below). The *E. coli* strains C43(DE3) (wildtype with respect to DctA) and IMW660 (DctA deficient) were grown in the presence (orange) or absence (gray) of fumarate. The bacteria contained plasmids encoding DcuS_Cys0_ variant with the respective Cys replacement mutations. Synthesis of the respective DcuS variants was induced by addition of 1 mM IPTG. Oxidative Cys CL was performed by the modified procedure with Cu^2+^ phenanthroline concentrations and temperature as described in experimental procedures, for bacteria grown with and without fumarate. The ratio of CL products to the total amount of DcuS was calculated after scanning the anti-DcuS Western blots and evaluating the band intensities in ImageJ software as described [13].

Oxidative Cys-cross-linking (Cys-CL) has shown that the TM2/PAS_C_-Linker plays an important role in the intramolecular signal transfer of DcuS [13]. Fumarate stimulation causes in the Linker a characteristic reduction in homo-dimerization whereas TM2 and the neighboring helix α1 of PAS_C_ are permanent homo-dimers in the absence and presence of fumarate. Here, oxidative Cys-CL was tested for representative residues of the TM2, the Linker and the α1-PAS_C_ regions in strain IMW660 that lacks DctA (Figure 2) and then compared (Table 1) to the oxidative Cys-CL in the parental strain (C43(DE3)) that is positive for DctA [13]. Conditions for testing were the same for both strains, including aerobic growth to exclude production of DcuB which is synthesized only under anaerobic conditions [3,33]. The bacteria produce plasmid-encoded variants of DcuS_Cys0_ that is deficient of all native Cys-residues [12,13]. In the variants individual amino-acid residues were substituted by single Cys residues. Oxidatively induced Cys-CL was analyzed in the bacterial cells, using the membrane-permeant copper(II)-(1,10-phenanthroline)_3_, or ‘Cu^2+^ phenanthroline’, as an oxidant [13,34] (Figure 2). DcuS and derivatives were visualized in non-reducing SDS-PAGE and Western blotting with DcuS specific antisera. The Western Blot shows DcuS monomers (apparent M_r_ ~55 kDa for His_6_-DcuS) clearly separated from cross-linked DcuS homo-dimers (apparent M_r_ ~170 kDa) (Figure 2D, Figure S1). The CL efficiency was calculated from the relative levels of cross-linked and free DcuS as described in [13].

Oxidative Cys-CL was tested in the time-resolved manner with increasing time for the CL-reaction. The modified procedure, using low temperature and low concentration of the oxidant for CL, has been shown to provide increased resolution of the CL-reaction [13]. First, residues Gly190 and Gly194 were tested (Figure 2A) representing residues with a response characteristic of TM2. DcuS(G190C) and DcuS(G194C) show in the strain lacking DctA an immediate and rapid increase of the CL efficiency. The final CL yields were obtained in less than 5 min. This response is nearly identical in the absence and the presence of fumarate. When the same experiment was performed earlier in the bacteria containing DctA [13], the kinetics of the labeling was very similar to the DctA-deficient strain. Therefore, the CL efficiency in TM2 is not affected by the presence of fumarate both in the presence and the absence of DctA (Table 1).

Next, the response of the TM2/PAS_C_-Linker region was tested (Figure 2B) by studying the response of the Ile208 and Leu209 residues. Both variants DcuS(I208C) and DcuS(l209C) show virtually no CL reaction in the absence of fumarate, and fumarate caused only a very low CL response for the DcuS(I208C) variant, whereas DcuS(L209C) did not react for the complete time. The response is clearly different to that of the wild-type [13]: The wild-type with DctA was in the CL response only silent in the presence of fumarate, whereas both residues showed a significant increase in the Cys CL reaction when fumarate was present [13]. Table 1 compares the rates of CL increase for the wild-type and the DctA deficient strain. The data high-light the complete loss of CL in the Linker region under DctA deficiency, and therefore a response typical for fumarate activation even though fumarate was absent.

In variant DcuS(R224C) where the Cys replacement is located in the α1 of PAS_C_, the CL reaction showed a slight increase both without and with fumarate in the the DctA deficient strain (Figure 2C). The CL efficiency was only slightly decreased compared to the DctA proficient state, both in the presence and absence of fumarate ([13] and Table 1). 

Overall, there is a clear change in the CL efficiency in the TM2/PAS_C_-Linker when DctA is missing compared to the wild-type as summarized in Table 1. The response in the DctA deficient strain resembles very much the response in the fumarate activated state of the wild-type (DctA proficient state) even when no fumarate is present. This effect of DctA deficiency is not seen for the TM2 region, and only to a small extent for α1 of PAS_C_. The data indicates that DctA deficiency causes conversion of the TM2/PAS_C_-Linker into the fumarate activated state even in the absence of fumarate, or vice versa that adjusting the fumarate negative or fumarate responsive state requires DctA. 

### 3.3. Modelling of DcuS and DctA

**Figure 3:**
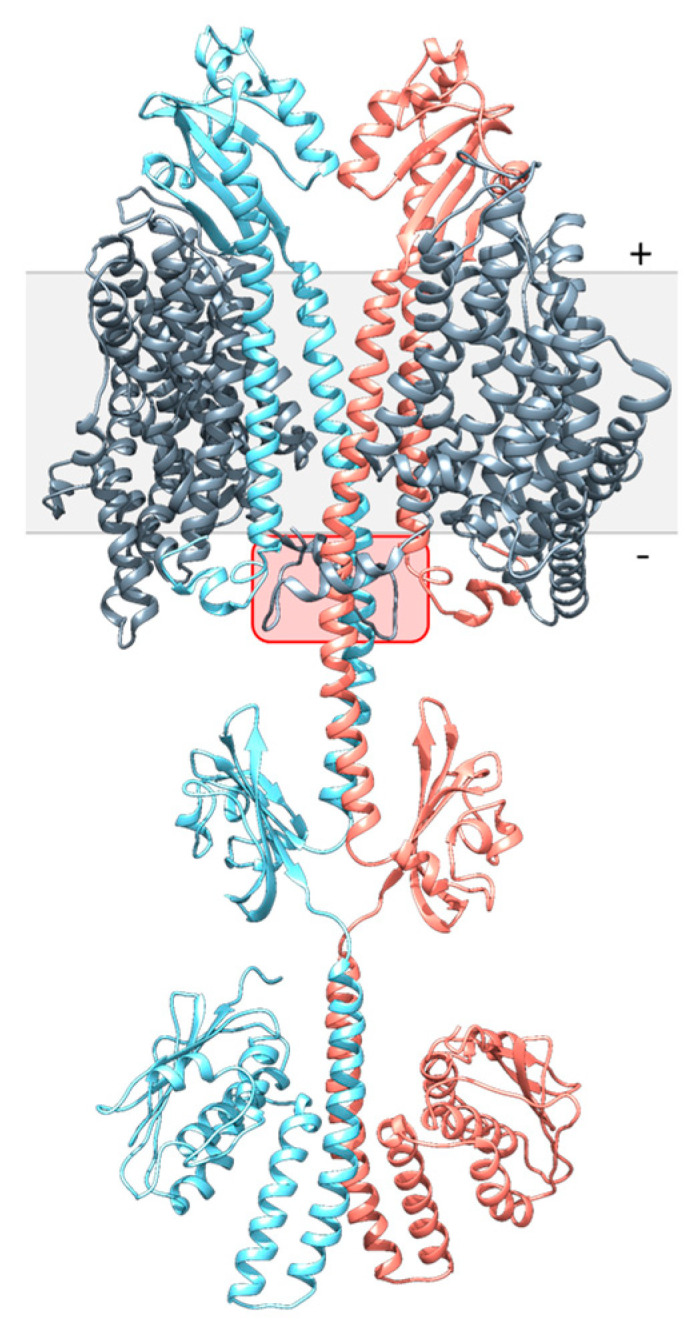
Interaction model between full-length DcuS and DctA. DcuS-DctA interaction was assembled manually from full-length DcuS and DctA (below). The interaction between DctA-H8b and DcuS-Linker is highlighted in the red box. From the DctA_3_ trimer only the interacting monomer is shown. Full-length DcuS was assembled from the *N*-terminal coil, TM1, PAS_P_, TM2, Linker, PAS_C_ and kinase domains [10,11,30], or ab initio using I-TASSER [29]. TM1, TM2, and the Linker were modeled manually in UCSF Chimera [25]. The kinase domain was modeled by I-TASSER, using the structurally similar kinase domain HK853 of *Thermotoga maritima* [29,30]. The individual structural models were loaded collectively into the molecular modeling program UCSF Chimera and assembled manually [25]. For the continuous α-helix connecting PAS_P_ and PAS_C_, biochemical CL data were available that identified residues involved in the homodimerization interface [12,13]. + and − represent the positive (periplasmic) and negative (cytoplasmic) sides of the membrane.

DcuS and DctA were modeled by homology modeling. DcuS was assembled from structures of the individual domains (*N*-terminal coil, TM1, PAS_P_, TM2, Linker, PAS_C_, and kinase domains) (Figure 3) using structures of close homologs CitA-PAS_P_ of *Klebsiella pneumoniae* and CitA-PAS_C_ of *Geobacillus thermodenitrificans* [10,11]. The kinase domain was modeled by I-TASSER from the kinase domain of sensor kinase HK853 of *Thermotoga maritima* [29,30]. TM1, TM2, and the Linker were modeled manually, and the *N*-terminal coil ab initio. After assembly [25], data from the dimeric PAS_P_, PAS_C_, and kinase domains were used to model DcuS as a dimer, and for the α-helix connecting PAS_P_ and PAS_C_, the residues involved in homo-dimerization [12,13] were used as dimerization sites.

The structural model of DctA represents a homology model derived from Glt as a template [35,36]. Glt from *Thermococcus kodakarensis* is a sodium-dependent L-Asp transporter and orthologous to DctA. Helix 3b and H8b, which are present only in DctA [21] were modeled ab initio.

Figure 3 shows a model for dimeric DcuS and monomeric DctA in the DctA × DcuS complex. DctA is presented as a monomer for clarity. The DctA × DcuS complex is based on the putative contact region between DctA H8b and DcuS TM2/PAS_C_-Linker suggested in earlier work by biochemical evidence [16,21]. The other parts of DctA and DcuS were modeled manually with the precondition to avoid collision sites between DctA and DcuS. The model shows an interaction site for H8b of DctA with the linker of DcuS in addition to interaction regions in the transmembrane and periplasmic domains of DcuS. TM2 is shielded from DctA by TM1, and in the model of Figure 3 TM1 serves as a major interaction surface for DctA. The interaction studies with truncated constructs show that DctA-H8b is essential for the DcuS-DctA interaction [16], which can be explained by the present model. Overall, structural modeling allows to merge biochemical and functional data for DcuS and DctA in a hypothetical complex and to predict sites for functional interaction in the Linker and H8b interaction in addition to other contact sites.

### 3.4. Structural Modelling of the DcuS Linker and DctA H8b Interaction

**Figure 4:**
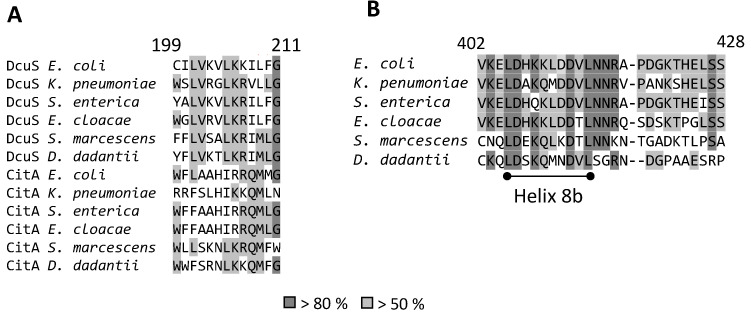
Multiple sequence alignment of the Linker regions of DcuS and CitA (**A**) and DctA H8b (**B**). The full length DcuS, CitA, and DctA sequences were obtained from UniProt [31] and aligned by the bioinformatics tool Unipro UGENE [38] using the MUSCLE algorithm in default settings [32]. (**A**) showing the Linker of DcuS and CitA, (**B**) showing DctA H8b region. The conservation levels are indicated for 50% (light grey) and 80% (dark grey).

The interaction model shows that DcuS can be accessed by the C-terminal H8b of DctA which approaches the TM2/PAS_C_-Linker of DcuS (Figure 3). The Linker sequence (Figure 4) reveals characteristics of some amino acid residues that could be important for interaction with H8b (Figure 4). Several residues are conserved in DcuS to higher extents than in the paralogous citrate CitA sensor kinases (Figure 4A). CitA constitutes together with DcuS the CitA family of sensor kinases [3,37], but CitA requires in contrast to DcuS no transporters as co-regulators [38] which suggests that residues involved in the Linker–H8b interaction of DcuS × DctA are not conserved in CitA. The L-X_3_-L-X_3_-L sequence of the Linker represents a homo-dimerization interface of DcuS [13] and should not be accessible to other proteins including H8b. Consequently, the underlined residues of the Linker C_199_I***L***V_202_K_203_V_204_***L***K_206_K_207_I_208_***L***FG_211_ (homo-dimerizing L-X_3_-L-X_3_-L sequence in bold italics) should remain for interaction with H8b. Among these, residues K203 and K207 are the most promising residues for interaction, as these residues are located on the opposite side of the linker-homodimerization interface. K203 showed moderate conservation of the positive charge for DcuS in the multiple sequence alignment, and this tendency is considerably enhanced in K206 and K207 (Figure 4A). The other residues of the Linker on the averted side (C199, I200, V202 and F210) are either not characteristic for protein-protein interaction, or in the border region of the homo-dimerization interface. Overall, based on chemical and structural properties, the L-Lys residues K203, K206, and K207 of the Linker are candidates for the interaction with DctA H8b, in addition to their role in providing positive charge according to the positive inside rule [12,39].

In H8b of DctA (sequence L_405_D_406_-HKK-L_410_-D_411_D_412_V-L_414_), D406, D411 and D412 are qualified for interaction due to their chemical characteristics (Figure 4B). Structurally, D412 would be most promising for interaction due to its location on the opposite side of the hydrophobic LD-X_3_-L-X_3_-L motif [21]. The suggested residues accumulate positive charges in the Linker and negative charges in H8b region with the potential to form salt bridges. 

**Figure 5:**
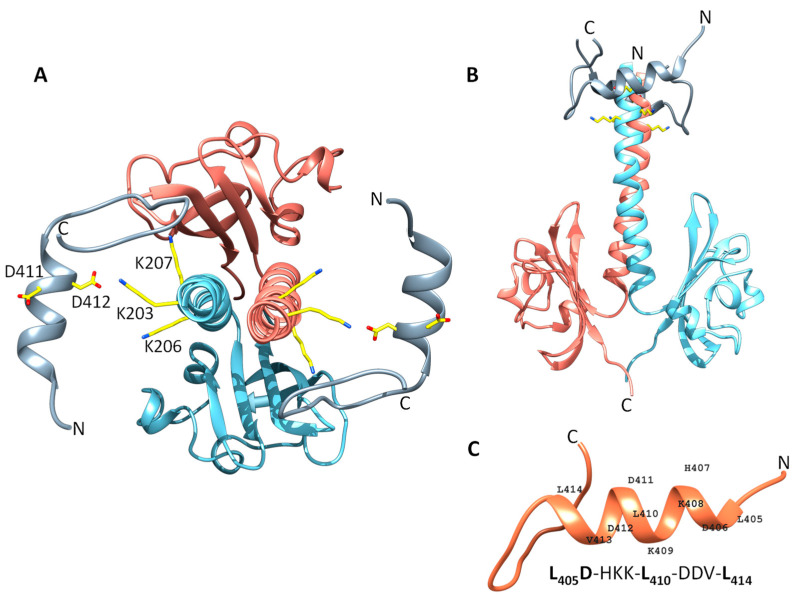
Scheme for the interaction between the DcuS Linker region and DctA H8b. (**A**,**B**) The interaction between DcuS Linker region (light blue and light red) and DctA H8b (dark gray) was modeled based on the selected residues, DcuS K203 and DctA D412. Both residues were selected based on conservation in the multiple sequence alignment (Figure 4), chemical characteristics, and biochemical data from previous work [13,16,21]. DcuS Linker-PAS_C_ model is modeled in dimeric form. Other residues important for interaction are annotated. The atoms are color coded as follows: carbon (yellow), oxygen (red), and nitrogen (blue). (**C**) Structural model of the H 8b region, wherein the residues of the α helical structure are annotated.

To model the interaction, the α-helical structures of the linker PAS_C_ and H8b were fitted into the cleft of DcuS by optimizing the structural arrangement and interhelical interaction of the residues. Modelling was performed in the molecular modelling program UCSF Chimera and for simpler presentation DcuS(K203) in the TM2/PAS_C_-Linker and DctA(D412) in H8b is shown (Figure 5). In the model DcuS(K203) and DctA(D412) are distant by approx. 2.6 Å which is compatible with the formation of a salt bridge. Alternatively, DcuS(K206) and DcuS(K207) can be arranged in positions for interaction with D412 of H8b. DctA(D411) can also be used for interaction with the L-Lys residues, which requires a different angling of H8b and moving the hydrophobic LD-X_3_-L-X_3_-L motif of H8b towards the membrane [21]. Overall, H8b of DctA can be positioned in the cleft of DcuS close to the TM2/PAS_C_-Linker which brings the acidic L-Asp residues of H8b in a position for salt-bridging with the basic L-Lys of the TM2/PAS_C_-Linker. Overall, the modeling provides data to test the interaction through structural and functional approaches.

## 4. Discussion

### 4.1. DctA as a Bifunctional Protein: Function as a Transporter and as a Structural Co-Regulator of DcuS

#### DctA as a Transporter for C4DCs

**Figure 6:**
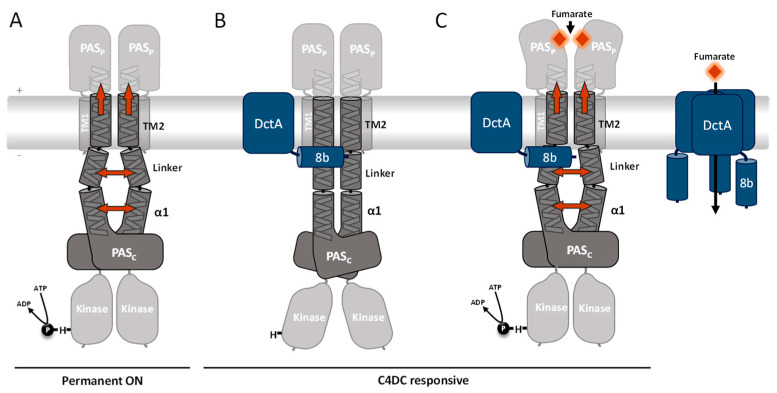
Schematic presentation of DcuS signal transduction and activity state in the absence (**A**) or presence of DctA (**B**,**C**), and of free DctA_3_. (**A**) In absence of DctA homo-dimerization of the TM2/PAS_C_-Linker is not stabilized, resulting in destabilization and partial loss of the dimeric state in the Linker and α1 of PAS_C_ (orange arrows), and shift of the TM2 piston [13]. The tilting movement and restructuring in the Linker is transmitted to PAS_C_ and the kinase, which adopts an active conformation and gets autophosphorylated (ON state). (**B**,**C**) DctA interacts via helix8b (H8b) with DcuS in the Linker region which stabilizes the Linker and the TM2/TM2′ double helix in the cytoplasmic arrangement. In this form, DcuS exists in the C4DC (fumarate)-responsive state (DctA × DcuS sensor complex). (**B**) Without fumarate (orange rhomb) the DctA stabilized DcuS is silent, and the kinase is inactive. (**C**) In the presence of fumarate, the fumarate binds to PAS_P_ which compacts and triggers a TM2 uplift which is transmitted to the TM2/PAS_C_-Linker. The shift of the Linker to the membrane/water interface causes rearrangement of the Linker. The structural reorganization is transferred to PAS_C_ and the kinase resulting in activation as described for (**A**). Domains, whose representative positions were subject to changes in the oxidative Cys-CL upon fumarate activation (see text) are colored opaque; other domains are grayed. Under induced conditions DctA is present in large excess to DcuS, and more than 95% of the DctA is present in the free state (right part of (**C**)) whereas essentially all DcuS is complexed (see text for details). Free DctA functions as C4DC uptake transporter). DctA prevails as a supposed trimer DctA_3_, DcuS is a permanent dimer (see text for details). It is not known whether DctA in the DctA × DcuS complex is transport active.

The primary function of DctA is the uptake of C_4_-dicarboxylates [40]. The absolute contents of DctA in membranes of *E. coli* amount to 20.3 pmol/mg protein of trimeric DctA_3_ compared to 0.45 pmol/mg of dimeric DcuS_2_ under fumarate inducing conditions [41]. For DctA a trimeric state is assumed based on the structure of the ortholog Glt [35], whereas for DcuS a dimeric state was experimentally determined [6]. Therefore, DctA_3_ is in nearly 46-fold molar excess over DcuS_2_ when a DctA_3_ × DcuS_2_ complex is assumed, or in a 23-fold excess assuming a DctA_3_ × DcuS_2_ × DctA_3_ complex. Hence, more than 95% of DctA is in the free state as a transporter (Figure 6). DcuS, on the other hand, is found essentially only in the complexed state [42]. Numerical observation of free DctA versus presence of DctA × DcuS complexes is confirmed in *E. coli* cells by fluorescence microscopy [43]. DcuS is localized close to the cell poles when expressed at low and physiological levels [42,43]. DctA, however, is scattered in the membrane when expressed in physiological levels, as the free form dominates under these conditions. When, however, the level of DcuS is increased artificially, the amount of DctA × DcuS increases and DctA is attracted and visible at the cell poles [43]. The data confirm that DcuS prevails in situ in complex with DctA, but there is an excess of DctA which is dispersed over the cell membrane. Under anaerobic conditions the C4DC/succinate antiporter DcuB is produced and takes over the function of DctA [19,20].

### 4.2. DctA as a Structural Co-Regulator of DcuS and Formation of the DctA × DcuS Sensor Complex

For the function of DctA in the DctA × DcuS sensor complex a coherent picture emerges (Figure 6). The DctA × DcuS sensor complex contains binding sites for C4DC in DcuS [7,9] and DctA [40,44] (Figure 6B,C), which raised the question for the site that senses the regulatory signal. However, characteristic differences in substrate specificity for some C4DCs when transported by DctA (or DcuB) compared with specificity in DcuS-dependent regulation showed that sensing occurs in DcuS [8,17,18,40,44]. Additionally, the substrate specificity of sensing can be altered by point mutations in the C4DC-binding site PAS_P_ of DcuS [17,41,45]. Finally, the *K_M_* values for transport of the C4DCs by DctA and DcuB [19,40,44] are by more than one order of magnitude lower than the apparent *K_M_* values for regulation of DcuS [8]. In summary, the data proves that sensing occurs in DcuS, but not in the transporters.

Presence of a transporter in sensory complexes promotes the idea that the transport process provides the regulatory signal to the attached sensor [46]. However, transport inactive point mutants of DctA [17] or DcuB [15] retained their capacity for coregulation of DcuS. Additionally, there are substrates such as maleate for DctA × DcuS [17], or maleate and L-tartrate for DcuB × DcuS [18], or citrate for a DcuB × DcuS* variant [18] that are not transported but have full regulatory capacity. Therefore, neither the transport nor the transport process provides the regulatory signal for DcuS. The experiments also provide strong arguments against secondary or allosteric regulatory sites in DctA and DcuB when acting as co-regulators of DcuS. 

Present and previous work shows that DctA (or DcuB) have a structural role in the sensor complexes, rather than in substrate or flux sensing. The present study provides in addition first information on functional aspects of co-regulation by demonstrating that DctA adjust the TM2/PAS_C_-Linker region to the fumarate sensitive OFF state. The data allows a conclusive model on the function of DctA in the DctA × DcuS (or DcuB × DcuS) sensor complexes (Figure 6C). According to this scheme, the C4DCs are perceived by PAS_P_, which causes uplift of α6-PAS_P_ and transmembrane signaling by the TM2/TM2′ piston-type displacement (Figure 6C). The TM2/PAS_C_-Linker transmits a tilting motion to the PAS_C_ and kinase domains. DctA-H8b intervenes at this point by interacting with the Linker, converting it to the OFF state, which then requires fumarate for activation. Obviously, the Linker controls the functional state of DcuS, and this adjustment requires interaction of DctA. The Linker region is supposed to adopt a highly similar structural arrangement in the fumarate activated ON state of the DcuS × DctA complex as in the DctA deficient ON state of DcuS (Figure 6A,C). The ON state represents the structural ground state which arises spontaneously when the structuring or stabilizing effect of DctA-H8b is missing. In the absence of DctA the TM2/PAS_C_-Linker is permanently in the functional OFF state (Figure 6A). Thus, DctA is a structural adaptor that places the Linker region in the fumarate-sensitive ground state. Remarkably this sensitizing is not required for the homologous citrate sensor CitA, which functions without co-regulating transporters [37]. It can be speculated that the difference is related to the large difference in substrate affinity of DcuS (app. *K_M_* about 2 mM for C4DCs) and CitA (*K_d_* about 1 µM for citrate).

## Figures and Tables

**Table 1:** Relative rate of Cys CL of DcuS with Cys replacements in the TM2 and the Linker regions of the wildtype and the *dctA* mutant. The rates are derived from the increase in the CL in the period of time showing the highest rate (0 to 3 min for DcuS(G190C) and DcuS(G194C) variants, and 10 to 20 min for variants DcuS(I208C), DcuS(L209C) and DcuS(R224C). Data are derived from Figure 2, and from the experiments in [13] for the wildtype (Wt). – and + Fum stands for absence and presence of fumarate.

Cys Cross-Linking Increase (% of CL per min)				
DcuS variant	*E. coli* C43(DE3) (Wt)		*E. coli* IMW660 (DctA^−^)	
	− Fum	+ Fum	− Fum	+ Fum
G190C (TM2)	9.2	9.7	7.7	6.5
G194C (TM2)	7.8	7.1	7.3	7.7
I208C (Linker)	2.6	-0.1	0.0	0.0
L209C (Linker)	2.8	1.4	0.0	0.0
R224C (PASc)	3.5	2.7	2.1	1.0

## Data Availability

Data are contained within the manuscript or the Supporting information. Quantitative data from Western blotting (ImageJ) are available from the corresponding author on request.

## Supplementary Materials

The following are available online at https://www.mdpi.com/article/10.3390/microorganisms9071397/s1, Figure S1: Detection of DcuS TM2 (G190C, G194) (A) or Linker variants (I208C, L209C) (B). after CL of DctA deficient bacteria (IMW660) with Cu^2+^ phenanthroline. Table S1: Strains and plasmids used. References [47,48,49,50] are cited in Supplementary Materials.
