# Supplementary material for: Conversion of the Sensor Kinase DcuS to the Fumarate Sensitive State by Interaction of the Bifunctional Transporter DctA at the TM2/PASC-Linker Region
Source: Microorganisms. 2021 Jun 28;9(7):1397. doi: 10.3390/microorganisms9071397 (PMC8307970; doi:10.3390/microorganisms9071397)
Supplement: Supplementary file 1 [file microorganisms-09-01397-s001.zip › microorganisms-1238770-supplementary.pdf]

Figure S1

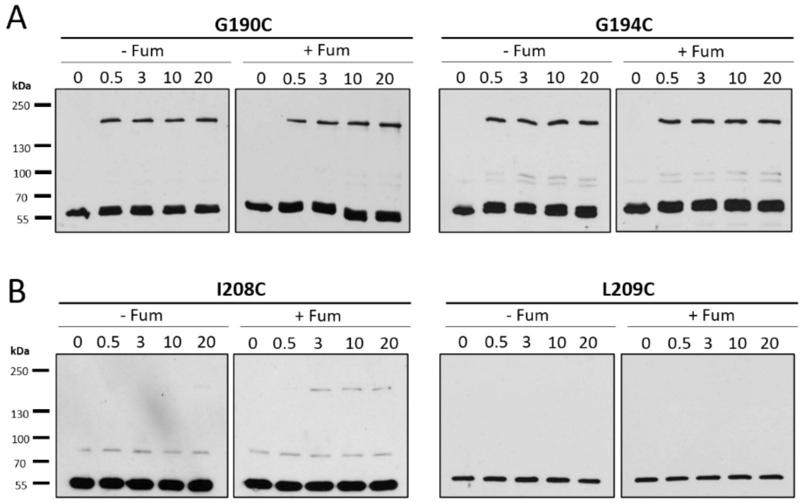

Figure S1. Detection of DcuS TM2 (G190C, G194) (A) or Linker variants (I208C, L209C) (B). after CL of DctA deficient bacteria (IMW660) with Cu^{2+} phenanthroline. Cross-linking was performed as described in [13]. CL-products were detected by chemiluminescence after non-reducing SDS-PAGE and Western blot. Scans of the X-ray films are shown. The reaction time is shown above the scans (0 to 20 min) and the absence (- Fum) or presence (+ Fum) of fumarate is indicated, as is the respective DcuS variant.

Table S1. Strains and plasmids used.

Strain	Genotype	Reference
BTH101	F$^-$, *cya-99, araD139, galE15, galK16, rpsL1 (Str^r), hsdR2, mcrA1, mcrB1*	[47]
C43(DE3)	F-, *ompT, gal, dcm, lon, hsdSB*(*rB*–*mB*–), λ(DE3 [*lacI lacUV5-T7p07 ind1 sam7 nin5*]), spontaneous mutation of BL21(DE3) to overproduce membrane proteins	[48]
JW4086	F-, *Δ(araD-araB)567*, *ΔlacZ4787*(::rrnB-3), *λ$^-$*, *rph-1, Δ(rhaD-rhaB)568, ΔdcuS751::kan, hsdR514*	[49]
JW3496	F-, *Δ(araD-araB)567*, *ΔlacZ4787*(::rrnB-3), *λ$^-$*, *ΔdctA783::kan, rph-1, Δ(rhaD-rhaB)568, hsdR514*	[49]
IMW660	C43(DE3) but *dcuS::FRT, dctA::FRT*	This study
pKT25	C-Terminal T25 protein fusion plasmid, pSU40 derivative, KanR	[50]
pKNT25	N-Terminal T25 protein fusion plasmid, pSU40 derivative, KanR	[50]
pUT18	N-Terminal T18 protein fusion plasmid, pUC19 derivative, AmpR	[50]
pUT18C	C-Terminal T18 protein fusion plasmid, pUC19 derivative, AmpR	[50]
pKT25-Zip	$_{T25}$Zip expression plasmid, pKT25 derivative, KanR	[47]
pMW336	His$_6$-DcuS expression plasmid but DcuS 199S-C471S, pET28-derivative (Kan^r)	[6]
pMW336	His$_6$-DcuS expression plasmid but DcuS 199S-C471S, pET28-derivative (Kan^r)	[6]
pMW429	$_{T18}$DcuS expression plasmid, pUT18C derivative, AmpR	This study
pMW1911	$_{T18}$PASc expression plasmid, pUT18C derivative, AmpR	This study
pMW1912	$_{T18}$PASc-Kinase expression plasmid, pUT18C derivative, AmpR	This study
pMW1913	$_{T18}$L-PASc expression plasmid, pUT18C derivative, AmpR	This study
pMW1914	$_{T18}$L-PASc-Kinase expression plasmid, pUT18C derivative, AmpR	This study
pMW1126	DctA$_{400-428}$-T25 expression plasmid, pKNT25 derivative, KanR	This study
pMW1915	$_{T18}$L^{-1}-PASc expression plasmid, pUT18C derivative, AmpR	This study
pMW1916	$_{T18}$L^{-2}-PASc expression plasmid, pUT18C derivative, AmpR	This study
pMW1917	$_{T18}$L^{-3}-PASc expression plasmid, pUT18C derivative, AmpR	This study
pMW1918	$_{T18}$L^{-4}-PASc expression plasmid, pUT18C derivative, AmpR	This study
pMW1919	$_{T18}$L^{-5}-PASc expression plasmid, pUT18C derivative, AmpR	This study

pMW858	$_{T25}$DctA expression plasmid, pKT25 derivative, KanR	This study
pMW1851	pMW336 but DcuS-I208C (KanR)	[12]
pMW1852	pMW336 but DcuS-L209C (KanR)	[12]
pMW2522	pMW336 but DcuS-G190C (KanR)	[13]
pMW2526	pMW336 but DcuS-G190C (KanR)	[13]
pMW2685	pMW336 but DcuS-R224C (KanR)	This study
